# Endovascular treatment of traumatic oronasal hemorrhage complicated with progressive acute epidural hemorrhage: A case report

**DOI:** 10.1097/MD.0000000000028654

**Published:** 2022-01-21

**Authors:** Zhinan Ye, Hanghuang Jin, Yuan Chen, Hailong Ji, Hao Xu, Yong Jin

**Affiliations:** a Department of Neurology, Municipal Hospital Affiliated to Taizhou University, Zhejiang, China; b Department of Neurosurgery, Municipal Hospital Affiliated to Taizhou University, Zhejiang, China.

**Keywords:** acute epidural hemorrhage, basilar skull fracture, endovascular treatment, middle meningeal artery, oronasal hemorrhage

## Abstract

**Rationale:**

Massive oronasal hemorrhage can induce shock and is life-threatening, and early endovascular treatment is the standard of care. Few studies have reported the use of endovascular treatment for acute epidural hemorrhage (AEDH). However, endovascular treatment of oronasal hemorrhage complicated by AEDH has not yet been demonstrated. Many patients with a low to moderate volume of oronasal hemorrhage complicated by AEDH choose conservative treatment but eventually undergo craniotomy due to increased intracranial hemorrhage.

**Patient concerns:**

A 32-year-old man presented to our hospital with traumatic oronasal hemorrhage complicated by AEDH after being hit by a blunt object.

**Diagnosis:**

Computerized tomography suggested progressive AEDH and multiple basilar skull fractures. Emergency cerebral angiography showed rupture of the right middle meningeal artery and a branch of the left maxillary artery causing AEDH and oronasal hemorrhage.

**Interventions:**

The patient underwent interventional embolization to treat the ruptured intracranial vessels.

**Outcomes:**

After 23 days, cranial computerized tomography showed remarkable absorption of the right frontal epidural hematoma, with the patient having a Glasgow Coma Scale score of 15.

**Lessons:**

This case provides a valuable treatment for patients with AEDH complicated with oronasal hemorrhage, early interventional embolization may be an effective treatment strategy to prevent further complications and ensure a good patient outcome.

## 1 Introduction

Surgery remains the main treatment strategy for hemorrhagic mass lesions in the brain, whether traumatic, spontaneous, or iatrogenic. Acute epidural hemorrhage (AEDH) is mainly caused by rupture of the middle meningeal artery. Patients who do not reach surgical indications usually undergo close neurological observation and repeated computerized tomography (CT) scans, and the incidence of hematoma enlargement ranges from 5.5% to 65%,^[1]^ which greatly increases the risk of unpredictable sudden neurological decompensation.^[2]^ Furthermore, AEDH is more likely to worsen in patients with oronasal hemorrhage caused by basilar skull fracture.

Emergency endovascular interventions are non-invasive and can effectively treat epidural hematoma enlargement without having to perform traditional craniotomy.^[3,4]^ Moreover, interventional embolization is an effective method for the treatment of oronasal hemorrhage caused by basilar skull fractures. Therefore, endovascular interventions may achieve good results in the treatment of AEDH complicated by oronasal hemorrhage caused by a basilar skull fracture. At present, there have been a few reports on endovascular treatment for AEDH, but no reports on endovascular treatment for oronasal hemorrhage complicated by AEDH.

Here, we present the case of a patient with posttraumatic oronasal hemorrhage complicated by AEDH, who was successfully treated with endovascular interventions during the early stage.

## 2 Case presentation

**Figure 1:**
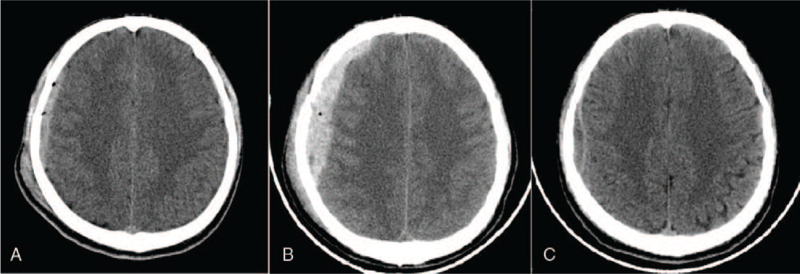
(A) First CT scan showing acute epidural hematoma in the right forehead. (B) Second CT scan, obtained at admission, showing that the hematoma had enlarged. (C) Follow-up CT scan obtained 23 days after embolization showing the hematoma had significantly reduced. CT = computerized tomography.

**Figure 2:**
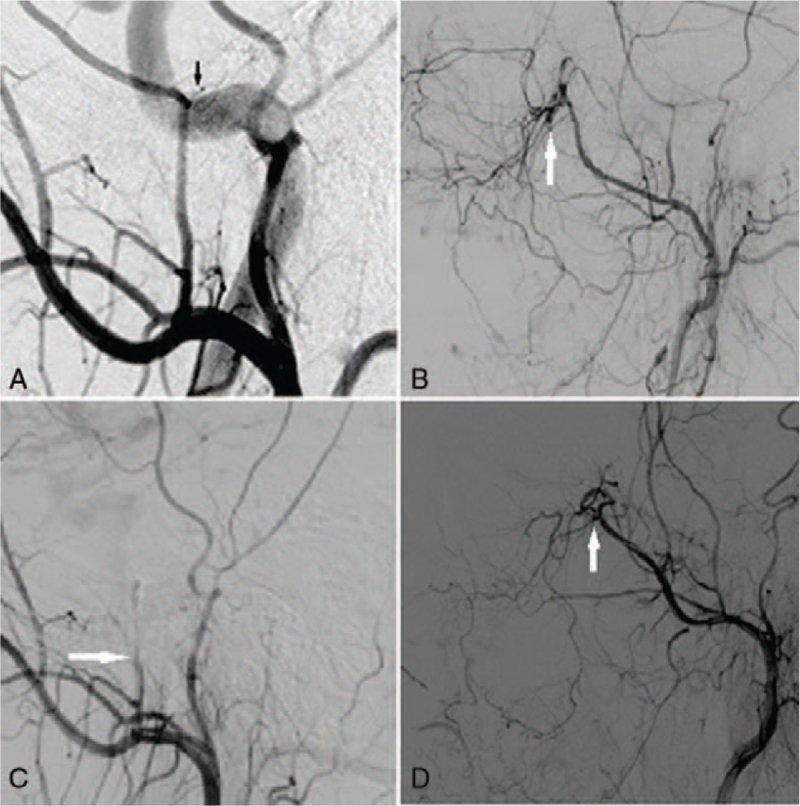
(A) Selective angiogram showing contrast medium exosmosis of right middle meningeal artery (black arrows). (B) Selective angiography showing smoke-like exudation of contrast medium from the distal branch of the left maxillary artery (white arrow). (C) Cerebral angiography showing right middle meningeal artery embolism (white arrow). (D) Angiography showing distal embolization from the left maxillary artery (white arrow).

A 32-year-old man was admitted to the Hospital on October 15, 2019, for head trauma and pain caused by an oronasal hemorrhage, which occurred immediately after being hit by a blunt object and lasted for 3 hours. CT suggested an epidural hematoma of approximately 10 mL (Fig. 1A). He was immediately transferred to the emergency room of our hospital for cranial CT, which suggested a right frontal epidural hematoma with a bleeding volume of approximately 25 mL (Fig. 1B), and multiple basilar skull fractures. The patient was lethargic, had bilateral nasal and mouth bleeding, and had a Glasgow Coma Scale score of 11 (E3V3M5). Because the patient was unmarried, the family members strongly requested that there be no critical situations, such as cerebral hernia, and did not consider surgery. Emergency cerebral angiography was performed under general anesthesia, which showed a ruptured right middle meningeal artery (Fig. 2A) and branch of the left maxillary artery (Fig. 2B), no obvious abnormalities were found in other cerebral vessels. Three spring coils (MicroVention model 100202HCSR-S-V) were used to embolize the ruptured middle meningeal artery, while the other 3 spring coils (MicroVention model 100202HCSR-S-V) embolized the ruptured maxillary artery branch (no suitable polyvinyl alcohol particles at that time). After interventional embolization, cerebral angiography showed that the distal ends of the right middle meningeal artery and left maxillary artery were no longer present (Fig. 2C and D). Postoperative head CT suggested that the right frontal epidural hematoma was similar to that observed in the previous CT scan. On November 7, cranial CT showed remarkable absorption of the right frontal epidural hematoma (Fig. 1C), with the patient having a Glasgow Coma Scale score of 15.

## 3 Discussion and conclusions

Hematoma evacuation is a regular treatment option for patients with AEDH who meet surgical indications. In the past, for the treatment of AEDH without surgical indication, either conservative treatment or hematoma evacuation could be performed after surgical indication, which may delay the effective treatment time. Recent studies have shown that angiography and intravascular intervention can be used to treat epidural hematoma or progressive epidural hemorrhage.^[5,6]^ AEDH is generally complicated by different degrees of skull fractures, skull base fractures, brain contusions, and lacerations. Oronasal hemorrhage caused by craniocerebral trauma often indicates skull base fracture. Skull base fracture is a common complication of AEDH that may easily lead to rapid and serious deterioration of the disease.^[7]^ Many studies have shown that endovascular treatment is effective for posttraumatic oronasal hemorrhage following traumatic brain injury.^[8–10]^ For patients with AEDH without surgical indication and complicated by massive oronasal bleeding, interventional embolization may be an effective treatment method.

An emergent craniotomy is indicated for an AEDH volume >30 mL, thickness >15 mm, midline shift greater than 5 mm, or clinical deterioration.^[2]^ However, patients with AEDH who have not reached the surgical indication are usually still under close neurological observation and repeated CT scanning to observe the condition, but the incidence of hematoma enlargement is often high, which not only greatly increases the economic cost and radiation exposure, but also carries the risk of unmeasured sudden nerve decompensation. AEDH patients with oronasal bleeding are more likely to deteriorate,^[9]^ and vascular interventions for those patients can detect ruptured intracranial vessels early on during trauma and allow early embolization, thereby reducing the risk of bleeding recurrence, persistent bleeding, or neurological function deterioration.^[6]^

Here, a patient with AEDH complicated by oronasal hemorrhage caused by a basilar skull fracture was treated with endovascular interventions in the acute phase. Cranial CT showed that epidural hemorrhage increased significantly in the early stage of trauma, suggesting that epidural hemorrhage continued to increase progressively. Angiography revealed a ruptured middle meningeal and maxillary artery, and the ruptured vessel was immediately treated with interventional embolization. After embolization, the epidural hemorrhage was gradually absorbed, oronasal bleeding stopped, and recovery was good, demonstrating that endovascular treatment is an effective and appropriate treatment strategy for oronasal hemorrhage caused by a basilar skull fracture complicated with arterial AEDH.

In conclusion, for patients with AEDH complicated by oronasal hemorrhage caused by a basilar skull fracture, early interventional embolization may be an effective treatment strategy to prevent further complications and ensure good patient outcomes.

## Author contributions

**Resources:** Hanghuang Jin, Yuan Chen, Yong jin.

**Writing – original draft:** Zhinan Ye, Hailong Ji, Hao Xu.

**Writing – review & editing:** Zhinan Ye, Yong jin.
